# Clinical Utility of the Sivelestat for the Treatment of ALI/ARDS: Moving on in the Controversy?

**DOI:** 10.1007/s44231-022-00012-5

**Published:** 2022-08-26

**Authors:** Qiongli Ding, Yi Wang, Chunbo Yang, Xiang Li, Xiangyou Yu

**Affiliations:** 1 Critical Medicine Center, The First Affiliated Hospital of Xinjiang Medical University, Urumqi, 830054 Xinjiang Uygur Autonomous Region China; 2 Xinjiang Medical University, Urumqi, 830054 Xinjiang Uygur Autonomous Region China; 3 Xinjiang Uygur Autonomous Region Institute of Critical Medicine, Urumqi, 830054 Xinjiang Uygur Autonomous Region China

**Keywords:** Sivelestat, Acute respiratory distress syndrome

## Abstract

Acute respiratory distress syndrome (ARDS) is a serious condition that can arise following direct or indirect acute lung injury (ALI). It is heterogeneous and has a high mortality rate. Supportive care is the mainstay of treatment and there is no definitive pharmacological treatment as yet. In nonclinical studies, neutrophil elastase inhibitor sivelestat appears to show benefit in ARDS without inhibiting the host immune defense in cases of infection. In clinical studies, the efficacy of sivelestat in the treatment of ARDS remains controversial. The currently available evidence suggests that sivelestat may show some benefit in the treatment of ARDS, although large, randomized controlled trials are needed in specific pathophysiological conditions to explore these potential benefits.

## Introduction

A cute respiratory distress syndrome (ARDS) is an acute inflammatory lung injury, associated with increased pulmonary vascular permeability, increased lung weight, and loss of aerated lung tissue [1]. Uncontrolled neutrophil-dominant inflammation and increased permeability of lung microvascular endothelium and alveolar epithelial cell layers are common pathophysiological features of ARDS, and clinically lead to nonhydrostatic pulmonary edema. Patients with ARDS develop severe damage to the lung in response to various insults including pneumonia, sepsis, trauma, burns, or acute pancreatitis. From severe acute respiratory syndrome (SARS) in 2003, to middle east respiratory syndrome (MERS) in 2012, to coronavirus pneumonia (COVID-19) in the winter of 2019, the incidence of ARDS in these three outbreaks was 20%, 20–30%, and 18–30%, respectively [2]. A number of ventilatory interventions, such as lower tidal volumes [3], higher positive end-expiratory pressure (PEEP) [4], and adjuncts such as prone positioning, neuromuscular blockade [5], and extracorporeal membrane oxygenation [6] for ARDS have been proposed. These supportive treatments that provide protective lung ventilation are designed to give patients a chance to repair their lungs. Although the development of therapeutic strategies such as protective mechanical ventilation technology has improved the mortality of ARDS patients, there is currently no effective drug for reducing the associated mortality.

## Neutrophil Elastase in the Pathogenesis of ARDS

**Fig. 1:**
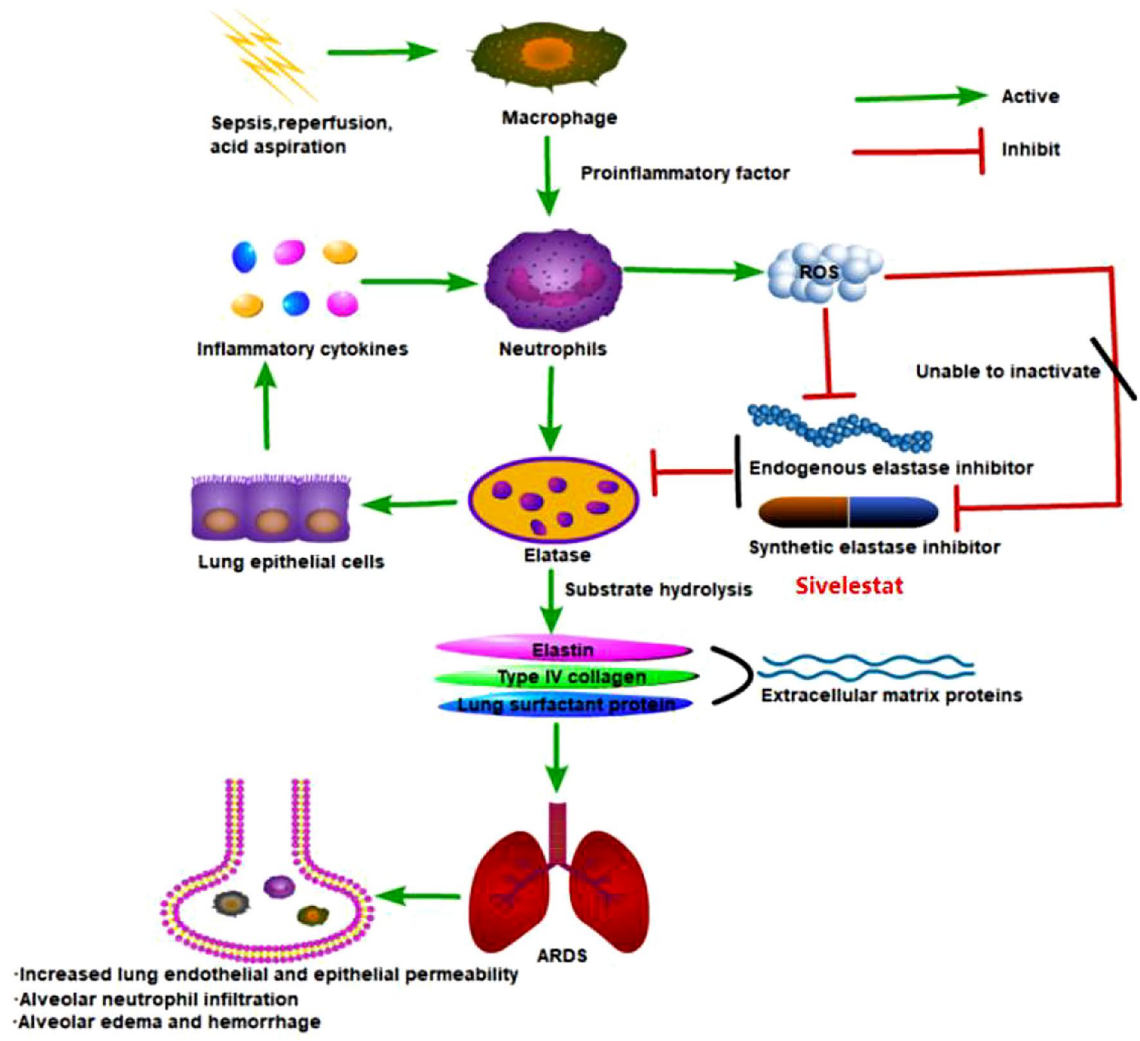
Possible role of neutrophil elastase in pathogenesis of ALI/ARDS

Neutrophil elastase (NE) is a serine protease produced by neutrophils. Its main physiological function is the degradation of phagocytosed foreign organic molecules within the cells. Extracellular neutrophil elastase is a highly destructive enzyme, capable of degrading a variety of extracellular proteins, including elastin, collagen, lung surfactant, and immunoglobulins. In addition to its proteolytic activity, neutrophil elastase is also known to induce the production of inflammatory cytokines [7] and mucin from epithelial cells. However, under physiological conditions, extracellular neutrophil elastase activity in the body is tightly regulated by endogenous protease inhibitors, such as α_1_-protease inhibitor. At inflammatory sites, the α_1_-protease inhibitor is inactivated by neutrophil-derived reactive oxygen species, thereby allowing extracellular neutrophil elastase to attack tissues. NE is not only an important damage molecule associated with ARDS, but also promotes the production of neutrophil chemokines and aggravates the inflammatory response. Therefore, inhibition of NE activity can prevent and alleviate ARDS. A possible role of neutrophil elastase in the pathogenesis of ARDS is shown in Fig. 1.

## Clinical Studies of Sivelestat

Sivelestat (SV) is a highly specific and systemically active NE inhibitor with a low molecular weight which acts into the intercellular space and works by inhibiting the activity of NE in ARDS [8]. Earlier studies in Japan have confirmed that the effects of sivelestat in reducing the permeability of pulmonary blood vessels [9], inhibiting mucus secretion in the epithelial layer [10], and decreasing the production of inflammatory cytokines such as IL-1β, IL-6, and TNF-α at clinically available concentrations [11] and protecting against postperfusion-induced lung injury [12]. In 2003, sivelestat entered clinical research due to the outbreak of SARS, which made sivelestat the first SARS treatment drug to enter clinical trials after the rapid approval channel was launched in China. Sivelestat, which undertake an important business for the first time, was actually a rising star at the time, as it was first licensed to manufacture in Japan in 2002 and became the global first drug for the treatment of ALI with systemic inflammatory response syndrome (SIRS). In the same year, a phase III clinical trial was conducted in Japan in which 230 ALI patients with SIRS were enrolled, and finally 221 patients were evaluated at the end point and found that compared with the low-dose group (0.003 mg/kg/h), the patients with SIRS who received high-dose group (0.16 mg/kg/h) had a higher proportion of moderate or significant improvement (71.7% vs. 55.6%), shorter mechanical ventilation duration (11d vs. 19d) and shorter ICU stays (16.5d vs. 29d) [13]. Since then, the drug has entered the field of vision. Some Randomized Controlled Trials (RCTs) have been conducted to validate the efficacy of sivelestat in ARDS (Table 1). The results showed that sivelestat therapy might play an important role on the PaO_2_/FiO_2_ level, while it had no significant effect on 28–30 days mortality, ventilation days, and ICU stays. The limitation of these studies are as follows: (1) the number of included studies was smaller than expected; (2) lack of uniform ARDS diagnostic criteria; (3) inevitable bias.

**Table 1:** Early clinical RCT studies of sivelestat in the treatment of ALI/ARDS

Study	Time	Group	Cases	Age	Disease status	Intervention	Baseline PaO_2_/FiO_2_ Ratio	Baseline APHACHEII Score
Kadio [14]	2004	Sivelestat	12	66	ARDS	0.2 mg/kg/h	142 (56)	19.9 (3.8)
		Control	12	62			155 (46)	20.2 (4.0)
Zeiher [15]	2004	Sivelestat	241	55.8	ALI	0.16 mg/kg/h	146.7 (57.1)	20.5 (6.8)
		Control	246	56.2			150.7 (59.0)	21.1 (7.2)
Tamkuma [16]	2004	Sivelestat	113	59.5	ALI	0.2 mg/kg/h	210.2 (85.1)	NR
		Control	108	56.1			187.3 (77.1)	
Endo [17]	2006	Sivelestat	13	NR	ALI	0.2 mg/kg/h	NR	NR
		Control	13					
Morimoto [18]	2011	Sivelestat	10	72	ALI	0.2 mg/kg/h	140 (53)	NR
		Control	12	74			96 (22)	

*NR* not reported

A possible explanation for the failure of other approaches may be that previous clinical trials included all patients who met the AECC diagnostic criteria, without the careful exclusion of patients with other diseases. Although these studies do not provide a general consensus on the clinical use of sivelestat, to date it is one of the few drug therapies for the treatment of ALI and ARDS.

In general, evaluating the effectiveness of pharmacotherapy for ARDS is difficult because of the nature of this multifactorial disease. Clinical findings and time courses vary among ALI patients, including those with ARDS, depending on the time after onset and the underlying diseases and injuries. In addition, the pathophysiological conditions and responses to treatment may be heterogeneous, and proving the pragmatic endpoint of landmark all-cause mortality rates in ARDS patients is difficult.

In the recent COVID-19, numerous reports outlined that a severe form of the disease in COVID-19 patients develops during a few days, which is often manifested as an acute lung injury/acute respiratory distress syndrome (ALI/ARDS), respiratory failure, heart failure or sepsis. And the proportion of deceased patients with ARDS was significantly higher than that of living patients (81% vs. 45%) [19]. The animal models of SARS-CoV and MERS-CoV showed that the significant levels of inflammatory and immune responses cause ‘cytokine storm’ and apoptosis of epithelial and endothelial cells. This is followed by an increase in vascular permeability and leakage, abnormal T-cell and macrophage responses, and ALI/ARDS that could eventually lead to death. We advocate the use of NE inhibitors such as sivelestat to alleviate neutrophil-induced damage in high-risk COVID-19 patients. Initiation of sivelestat will serve two strategic purposes; first, it will mitigate the damaging effect of neutrophil elastase on the lung connective tissue, and second, it will limit the virus spreading capabilities by preventing S protein proteolytic activation [20]. Clinical trials that reported positive outcomes of sivelestat treatment in patients with ARDS and ALI had recruited patients mainly with lung injury score (LIS) < 2.5. The study suggests that early administration of NE inhibitors to patients with lymphocytopenia and LIS < 2.5 May be of significant value in preventing disease progression, but current evidence to support the use of NEIs in ARDS induced by COVID-19 is lacking. In a study on 167 septic patients with ARDS and DIC, sivelestat was administered upon admission to ICU and continued for 5 days. The results showed that sivelestat improved lung injury score, PaO_2_/FIO_2_ ratio, DIC score, and ICU length of stay and survival rate when compared to the control group [21]. Based on its promising beneficial effects in underlying complications of COVID-19, this selective NE inhibitor could be considered as a promising treatment for better management of ALI/ARDS or coagulopathy in patients with COVID-19 [22].

A series of negative outcomes in RCTs have not made intensivists lose heart and enthusiasm for sivelestat. A large retrospective study of 4276 patients with ARDS conducted in 2017 found that actual mortality in the sivelestat group were 7.0%, 9.1%, and 8.9% lower than in non-sivelestat group at 30, 60, and 90 days, respectively; patients in younger age, absence of cancer, no need for haemodialysis and not using high-dose methylprednisolone were significantly correlated with treatment success; sivelestat might be more effective in Japanese patients or Asian ethnicities [23]. Similarly, a multicenter clinical trial showed that sivelestat can effectively improve the respiratory function of ALI/ARDS patients, and the ventilation days in sivelestat group was shortened by 3.5 days on average compared with the conventional treatment group [24]. In 2020, a recent study conducted in China also confirmed the efficacy of sivelestat in sepsis with ARDS, and it could significantly reduce the medical costs of patients in pharmacoeconomics [25]. With the application of sivelestat and other drugs, the treatment of inhibiting inflammatory overreaction in sepsis ARDS is entering a new era of targeted inhibition from the early non-specific inhibition of inflammation.

## Safety of Sivelestat

Although the promise of sivelestat is exciting, a number of questions remain. The timing and duration of sivelestat intervention may be crucial to its ultimate success. However, in the STRIVE study, some adverse events occurred, such as hypersensitivity, hepatobiliary disorders, blood and lymphatic system disorders, renal and urinary disorders. Although prespecified stopping guidelines were not met, a negative trend in long-term mortality prompted the DSMB to recommend suspension of enrollment and discontinuation of study drug [9]. To date, available clinical study data, including for the STRIVE study and the related postmarketing study, indicate no particular concerns regarding adverse events. The occurrence of adverse events must be confirmed in larger prospective RCTS and should be assessed over a longer period of follow-up.

Currently, clinical studies on sivelestat are mainly focused on inhibiting the activity of NE, but there are also studies that show that intravenous infusion of sivelestat in improving respiratory function in patients with cardiopulmonary bypass (CPB)-induced ALI is by inhibiting PMN elastase and IL-8 during the CPB process. This randomized, double-blind clinical trial shows that sivelestat has a good effect in the treatment of ALI after CPB [26, 27]. In addition, because different targets regulated by sivelestat may also play different roles, when to start or stop using sivelestat is also a clinical problem. A retrospective and observational study suggest that administration of sivelestat within 7 days of admission may improve the prognosis of patients with ALI/ARDS. To our knowledge, this is the largest study to evaluate the efficacy of sivelestat on ALI/ARDS [23]. Currently, in the latest publication of the "Sivelestat,Consensus of Clinical Experts", it is recommended that sivelestat be used within 24 to 72 h after onset of the disease, and the longest course of administration is 14 days, but the specific timing of discontinuation is unknown.

## Nonclinical Studies of Sivelestat

**Fig. 2:**
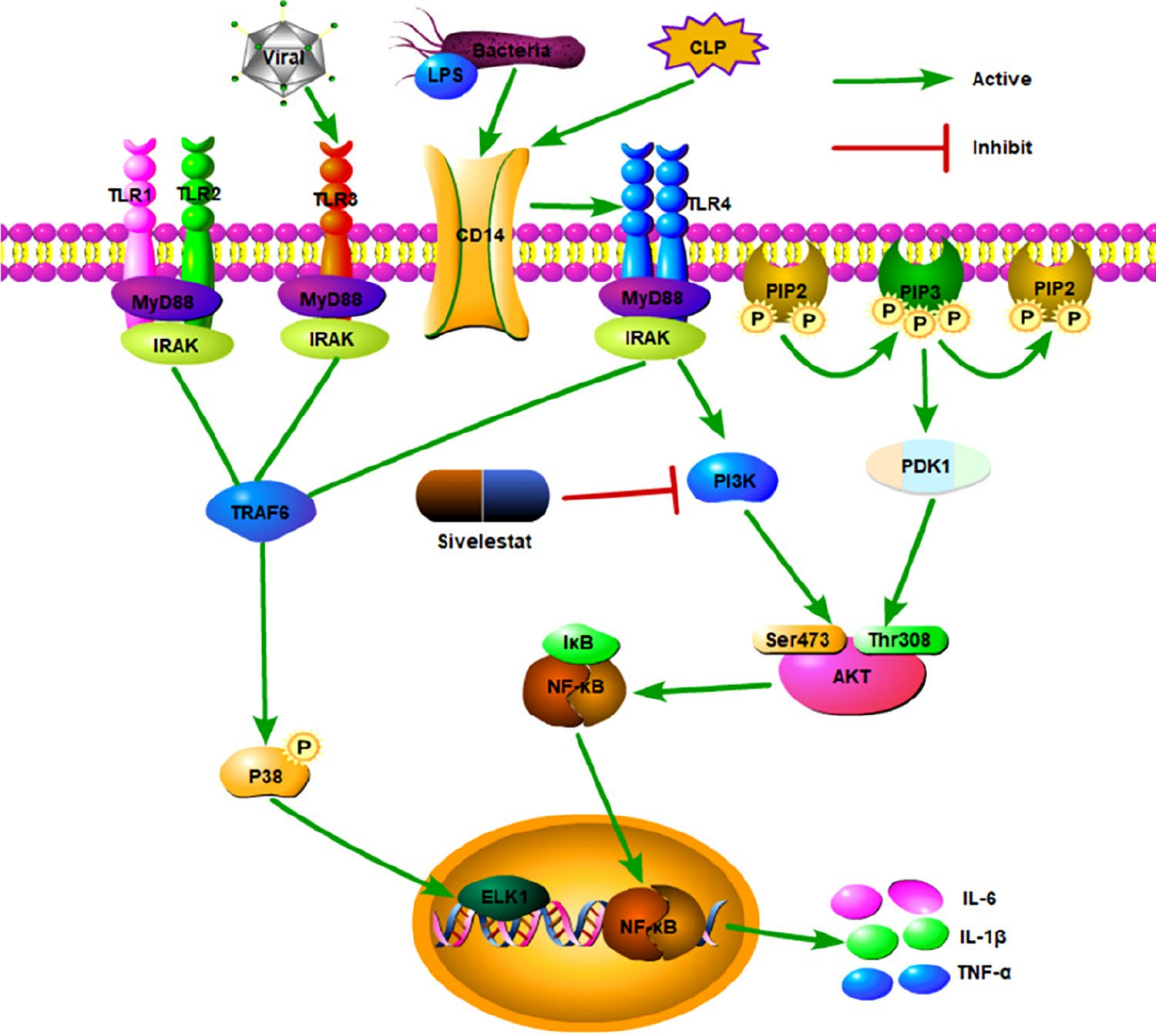
Effect of sivelestat on acute kidney injury in septic rats. Sivelestat might ameliorate acute kidney injury in septic rats by inhibiting PI3K/AKT signaling pathway

At present, it is believed that the immune inflammatory response in sepsis is a complex regulatory network involving multiple signal transduction pathways in the body. As illustrated in Fig. 2, the lipopolysaccharide (LPS) receptor-mediated signal transduction pathway is an important response mechanism of the body to bacterial invasion. After Toll-like receptor (TLR) recognizes a specific bacterial sequence LPS, it initiates a pro-inflammatory response through a positive feedback pathway, while activating Phosphatidylinositol-3-kinase (PI3K)/protein kinase B (protein kinase B, AKT) signal transduction pathway [28]. When PI3K/AKT is phosphorylated, it activates the downstream nuclear factor-κB (NF-κB) for nuclear translocation, initiates and regulates the transcription of inflammatory factor genes, and promotes the expression of inflammatory factors [29]. It is worth noting that the phosphorylation of AKT referring to PI3K/AKT pathway activation is enhanced in the lungs [30] and liver [31], but is weakened in the heart after the CLP procedure or LPS challenge. Consequently, both the blockade and activation of PI3K/AKT signaling transduction have been shown to improve outcome in septic shock. Our team conducted some basic studies on the target of sivelestat and demonstrate that the administration of the NE inhibitor, sivelestat, mitigates CLP-induced kidney injury, reduces inflammation, and suppresses the activation of the PI3K/AKT signaling pathway. Our study suggests that sivelestat has potential to attenuate sepsis-induced kidney injury, and high dose of sivelestat has better efficacy [32]. We found that CLP-enhanced renal PI3K/AKT phosphorylation was decreased by sivelestat. Moreover, the LPS induction of the phosphorylation of AKT is responsible for NF-κB activation in human renal mesangial cells [33], and the inhibitory effects of sivelestat on NF-κB signals have been previously reported. It is likely that PI3K/AKT signals are involved in the regulatory effects of sivelestat on the NF-κB pathway. Further in vitro experiments are thus being carried out by our group to study the underlying mechanisms [34].

## Conclusions

In conclusion, until the reemergence of ARDS in COVID-19, there are still limitations to treatment strategies, but the search for sivelestat, a potential stock that can inhibit ARDS, has never stopped or been absent. Although there is some evidence for the efficacy of sivelestat in specific clinical conditions, further studies, particularly randomized controlled trials, are needed to add to the current knowledge regarding the efficacy and safety of this agent in the management of ARDS. Although there are many therapeutic drugs aimed at blocking the progression of ARDS, which have important clinical application prospects, there is still insufficient evidence based on evidence-based medicine. In the future, more basic experiments or clinical studies should be carried out to identify patients who benefit from drugs, find effective indicators for efficacy evaluation, and establish more accurate treatment strategies.

## Data Availability

Not available.
